# Evaluation of Bait Acceptance and Immune Response in a Local Dog Population During an Oral Rabies Vaccination Field Study in Cambodia

**DOI:** 10.3390/tropicalmed11070204

**Published:** 2026-07-20

**Authors:** Bengthay Tep, Sochetra Hak, Nouv Sophorn, Sin Grandy, Ad Vos, Dim Kanan, Kinzang Dukpa

**Affiliations:** 1 Department of Animal Health—Veterinary Public Health, General Directorate Animal Health & Production, Phnom Penh 12352, Cambodia; 2 Ceva Tiergesundheit (Riems) GmbH, 17493 Greifswald, Germany; 3 Faculty of Veterinary Medicine, Royal University of Agriculture, Phnom Penh 12401, Cambodia; dkanan_vm@rua.edu.kh; 4 Regional Representation for Asia and the Pacific, World Organisation for Animal Health, Tokyo 113-0032, Japan; k.dukpa@woah.org

**Keywords:** rabies, oral vaccination, dog, SPBN GASGAS, bait acceptance, immunogenicity

## Abstract

(1) Background: Dog-mediated human rabies remains a serious public health problem in Cambodia, partially due to the high abundance of dogs. As most dogs are free-roaming and cannot easily be handled and restrained, the rabies vaccination coverage is very low. Oral rabies vaccination (ORV) could increase the vaccination coverage to levels required for interrupting the transmission cycle. (2) Methods: In this study, the bait acceptance and immunogenicity of a commercially available vaccine bait were tested in local free-roaming dogs in the northern part of Takeo province in Cambodia. (3) Results: In total, 75.4% (515/683) of dogs initially showed interest when offered an egg-flavored bait containing the SPBN GASGAS rabies virus vaccine. Subsequently, 89.9% of these dogs consumed the bait, and almost all of them (98.3%) were considered successfully vaccinated. Blood samples were collected from 50 dogs prior to vaccination. Eight of these fifty dogs tested sero-positive for rabies antibodies (ELISA) and were excluded from follow-up samples. Thirty and ninety days post-vaccination, 91.4% and 68.2% of the relocated vaccinated dogs tested sero-positive, respectively. (4) Conclusions: Bait acceptance and immunogenicity showed similar results as observed in studies with the same vaccine bait conducted in other countries. Hence, ORV seems to be a very promising tool for dog rabies control in Cambodia, which has a large owned but free-roaming dog population.

## 1. Introduction

Rabies is endemic in Cambodia, but the true incidence in both humans and animals is not known due to limited surveillance, especially in animals. Roughly between 100 and 200 rabies cases are reported in animals annually [1], and it is estimated that around 800 humans die of rabies [2]. The domestic dog is the most important reservoir species, and most reported cases originate from areas in close proximity to the capital, Phnom Penh. The relative abundance of dogs in Cambodia is extremely high. The dog-to-human ratio lies between 1:3 and 1:5 [2,3,4,5]. Localized mass dog vaccination (MDV) campaigns have recently been implemented in and around the capital, Phnom Penh, and almost all dogs encountered could be vaccinated by the parenteral route [6,7]. However, vaccination efforts also include catching dogs that could not be handled by their owners using large butterfly nets. This capture–vaccinate–release approach (CVR) has shown great potential in areas like Goa State, India, and Bali, Indonesia [8,9]. Unfortunately, this method is very labor-intensive and thus costly. Furthermore, it becomes less effective with time, as dogs become increasingly alert and cautious. Hence, it is becoming more difficult to catch dogs during subsequent MDV campaigns. Also, the risk of injuries for both vaccinators (bite incidents) and dogs should not be underestimated. Finally, these MDV campaigns, which have been successful in and around Phnom Penh with hundreds of people involved, including international volunteers [6,7], are difficult to implement nationwide.

Therefore, no systemic country-wide vaccination program—particularly in rural areas—has been conducted and consequently most dogs are not vaccinated against rabies. Most dogs are owned and only a small number of ownerless dogs are present, but the great majority of dogs are free-roaming and not easily accessible for vaccination. Considering the high number of free-roaming dogs inaccessible for direct parenteral vaccination in Cambodia and the labor force needed for CVR, oral rabies vaccination (ORV) has been suggested to increase accessibility of the local dog population for rabies vaccination and offer a more sustainable solution. Large-scale ORV campaigns have also been introduced in Namibia and neighboring Thailand and have shown a positive impact of this complementary vaccination tool in achieving a high vaccination coverage among owned and ownerless free-roaming dogs [10,11,12,13,14,15,16]. The study presented here aimed to investigate if the concept of ORV as practiced in neighboring Thailand would also be feasible in Cambodia. To determine if the available vaccine baits tested in Thailand would be acceptable to the predominantly owned dogs in Cambodia and capable of inducing a protective immune response, a small-scale WOAH-supported field trial was carried out. Hence, the objective of this exploratory and confirmatory field study was implementation-oriented. This study completes a series of similar bait acceptance and immunogenicity studies conducted in different settings worldwide, including Thailand, Indonesia, Navajo Nation [USA], Mexico, Namibia and Morocco [10,11,17,18,19,20,21,22]. The results of this study demonstrate that ORV in Cambodia is feasible and could contribute to the control of dog-mediated rabies by increasing the vaccination coverage of the dog population.

## 2. Materials and Methods

### 2.1. Study Area

**Figure 1:**
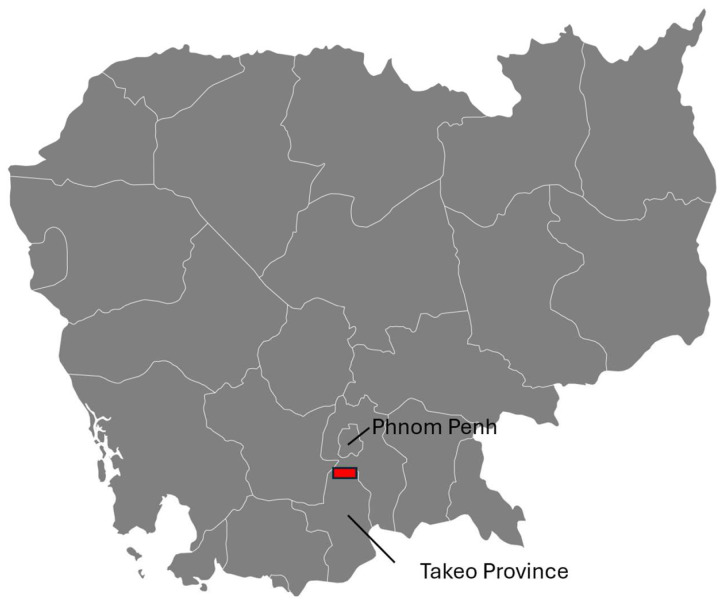
Study area (highlighted in red) in the northern part of Takeo Province, located 30 km south of the capital Phnom Penh in Cambodia.

The study was performed in a rural area south of Tonle Bati Lake, in the northern part of Takeo province, 30 km south of Phnom Penh, the capital of Cambodia (Figure 1). None of these communities had been involved in any dog population management interventions by local authorities or animal welfare organizations, including vaccination of dogs against rabies. Most houses are not fenced and animals are free to move within and beyond household premises.

### 2.2. Study Protocol

The sachet incorporated in the egg-flavored baits contained 3.0 mL SPBN GASGAS (10^8.1^ FFU/mL). This vaccine virus is a third-generation oral rabies virus vaccine, licensed in the EU for ORV in dogs, foxes and raccoon dogs [23]. The vaccine baits were stored in a freezer at −20 °C prior to the field study and kept in cool boxes during the field activities. During systematic coverage of the study area, every dog encountered was offered a single bait. The sample size for bait acceptance was based on the number of vaccine baits donated (*n* = 1000) and substantially exceeded the estimated minimum sample size calculated from the bait acceptance rate observed during previous field studies using the same vaccine bait.

If the dog’s owner could be identified, oral consent was obtained before the bait was offered to the dog. Observations were recorded electronically using the WVS App [24]. A recent risk assessment of this particular vaccine bait concluded that its use posed only a negligible risk to target and non-target species, including humans, as well as to the environment [25]. Nevertheless, basic safety precautions for vaccinators and dog owners were implemented (see: Rabitec Dog (ORV) Manual: https://www.rabiesfreestreets.com/ assessed 12 May 2026).

Data recorded included bait acceptance, consumption, sachet perforation, bait handling time, and vaccination success. Bait acceptance was defined as whether the dog showed interest in the bait through direct oral or nasal contact by sniffing or licking (‘interested’) or ignored the bait (‘ignored’). The latter category also included dogs that ran away when the bait was offered. Consumption was recorded based on whether the dog took the bait into its mouth and what proportion of the egg casing was consumed (<50%, >50% or 100%). Bait handling time was estimated based on the observed duration of bait manipulation (chewing) by the dog and categorized as very short [<10 s], short [10–30 s], medium [30–60 s] and long [>60 s]. The fate of the sachet was also recorded, specifically whether it was swallowed or discarded. Furthermore, it was recorded, where possible, whether discarded sachets had been perforated by the dog’s teeth. Finally, each vaccination attempt was evaluated by the recording staff member based on the previously described observations to determine whether the vaccine had likely been released into the oral cavity of the dog. Thus, during the bait acceptance study, dogs were classified as ‘vaccinated’ when the vaccine contained within the sachet was assumed to have been released into the oral cavity of the dog based on direct observations made during bait consumption. An “unknown” option was available for all variables if the outcome was not available, for example, when a dog moved out of sight while carrying the bait. Data recorded for each dog offered a bait included whether the dog was alone (‘single’) or accompanied by other dogs (‘multiple’), sex (male/female) and size (small/medium/large). The name of the recorder, location (GPS coordinates), and the time of observation were automatically stored for every data entry.

Fifty (50) dogs were selected for the immunogenicity study based on their accessibility (i.e., ease of handling) and whether they had accepted and consumed the bait. The minimum estimated sample size required was 35 animals, based on previous studies in Mexico and Indonesia that demonstrated an average seroconversion rate of 90% at 1 month post-vaccination using a similar vaccine dose [17,20] (10% margin of error). Because not all dogs can typically be relocated at subsequent sampling time points, additional animals were enrolled to compensate for potential losses to follow-up. After the purpose of the study and the associated procedures had been explained, oral consent was obtained from the owners prior to vaccination and blood sampling. For identification purposes, a photograph was taken of the dog together with its owner. A blood sample (B0) was collected immediately after bait consumption. Study animals were fed by their owners as usual, and no special diet was provided for the dogs during the study. Subsequent blood samples were collected approximately 30 (B1) and 90 (B2) days post-vaccination (dpv).

Blood samples were collected from the large superficial veins of the forelimb. The blood was transferred from the syringe into 5 mL uncoated blood collection tubes (S-Monovette, Sarstedt, Nümbrecht, Germany) and centrifuged at 3500× *g* for 15 min. Subsequently, sera were stored at −20 °C until laboratory analysis for the presence of rabies virus antibodies.

Serum samples were analyzed for the presence of rabies virus-binding antibodies (rVBA) using a commercial blocking enzyme-linked immunosorbent assay kit (BioPro Rabies ELISA, O.K. Servis BioPro, Prague, Czech Republic) following the manufacturer’s instructions and essentially as previously described [26]. In brief, microplate wells pre-coated with rabies antigen were incubated with diluted samples together with positive and negative controls. Following a washing step, a biotinylated anti-rabies antibody was added and incubated, after which the plates were washed and incubated with a streptavidin peroxidase conjugate. Following a final wash, the reaction was developed using a tetramethylbenzidine (TMB) substrate, which produced a blue coloration that turned yellow upon addition of the stop solution. The color intensity was measured at 450 nm, where a decrease in intensity relative to the negative control indicated a higher concentration of blocking antibodies in the sample. Results were expressed as a percentage blocking (PB) according to the manufacturer’s protocol, with a threshold of >40% PB defined as the cut-off for seropositivity.

All dogs included in both the bait acceptance and immunogenicity studies were clinically healthy and at least 3 months of age.

Statistical analyses (Chi-square test) were performed using GraphPad Prism 7 (GraphPad Software Inc., San Diego, CA, USA). Data entries recorded as “unknown” were excluded from percentage calculations.

## 3. Results

A total of 1238 data entries were recorded during the bait acceptance study; however, only 687 entries were considered valid and were suitable for data analysis. Several electronic records (*n* = 36) contained no information other than the name of the recorder, location and time. In addition, many forms (*n* = 515) containing contradictory data were excluded: (1) the bait was recorded as ‘ignored’ but the dog was subsequently classified as ‘vaccinated’ (*n* = 34), (2) the sachet was recorded as swallowed or its fate was classified as ‘unknown’ but nevertheless reported as perforated (*n* = 321), and (3) the sachet was recorded as not perforated, but dog was reported as successfully vaccinated (*n* = 160). This strict selection process had no impact on validity of the calculated bait acceptance rate. Previous bait acceptance studies using the same bait had demonstrated acceptance rates ranging from 65.0% to 80.0%. Assuming an average bait acceptance rate of 72.0% and a 10% margin of error, the calculated minimum sample size was 77 animals.

**Figure 2:**
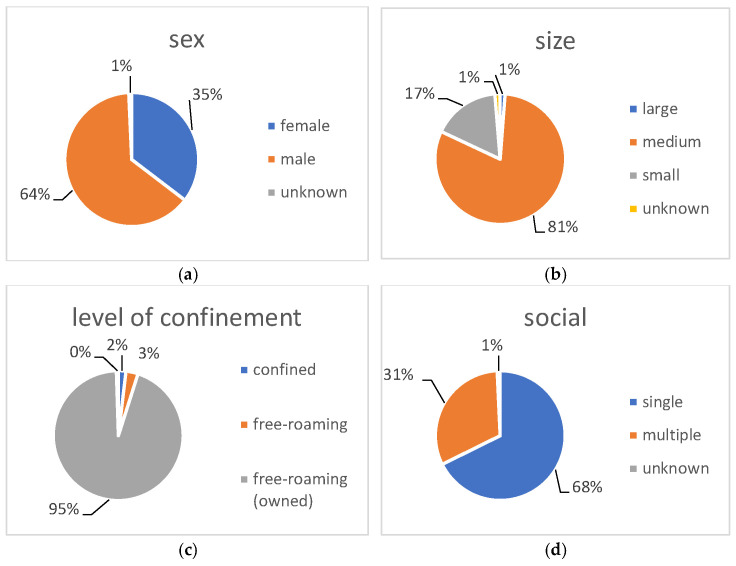
General description of the dogs offered a bait during the acceptance study; (**a**) sex (male or female), (**b**) size of dog (small, medium or large), (**c**) level of confinement (restricted [e.g., garden] or free-roaming) and (**d**) if dog was alone (single) or together with other dogs (multiple) when offered a bait.

General characteristics of the dogs included in the analysis are summarized in Figure 2. Most dogs offered a bait were medium-sized owned adult male dogs that were alone (‘single’) and free-roaming.

**Figure 3:**
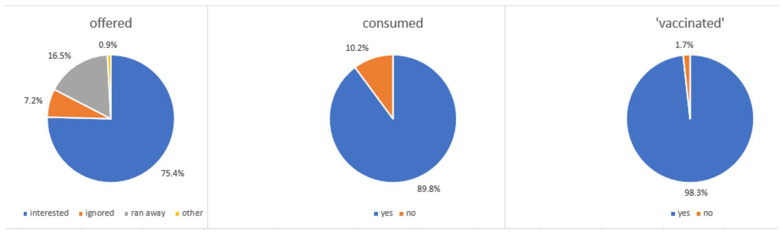
Bait acceptance and subsequent consumption—and vaccination rate as determined by release of the vaccine in the oral cavity of the dog through bait consumption. The consumption rate is based on the dogs showing interest and the vaccination rate is based on the animals that actually consumed the bait. Observations with ‘unknown’ response are not included.

Overall, 75.4% of the dogs showed interest in the baits offered and 89.8% of these dogs consumed the bait. Of the dogs that accepted and subsequently consumed the bait, 98.3% were considered vaccinated (Figure 3). In total, 451 (66.4%) of the dogs approached and offered a bait were considered vaccinated, whereas 228 (33.6%) were not, excluding 8 dogs with an unknown outcome. Of these 228, 113 (49.6%) ran off immediately when a bait was offered, 49 dogs (21.5%) ignored the baits offered and 52 (22.8%) showed interest but did not consume the bait.

Table 1 summarizes the proportion of the bait consumed, the time required for bait consumption, whether the sachet was perforated or not, and whether it was discarded or swallowed, including the associated vaccination rate. Testing for statistical significance (contingency testing using the Chi^2^-square test) could not be performed because the assumptions for the test were not met.

Additional information recorded on bait interest, bait consumption and vaccination success for each dog, including date and time of bait offering, whether the dog was alone or not (social), sex and size, is summarized in Table 2.

A significant effect of the factor ‘date’ was observed on the proportion of dogs that showed interest (*p* < 0.0001) and consumed (*p* < 0.0001) the baits offered (Chi^2^ test). However, these two responses were not linearly correlated (R^2^ = 0.0546); a high proportion of dogs showing interest in the bait on a given day was not always associated with a high consumption rate on that same day. Dogs showed the lowest level of interest in the bait during the early morning. Dogs that were alone (‘single’) showed interest in the bait more frequently than dogs that were in groups; however, the proportion of dogs that consumed the bait was higher among dogs in groups. No differences in bait acceptance or subsequent vaccination rates were observed between male and female dogs. Smaller dogs accepted and consumed the baits significantly more than larger dogs.

**Figure 4:**
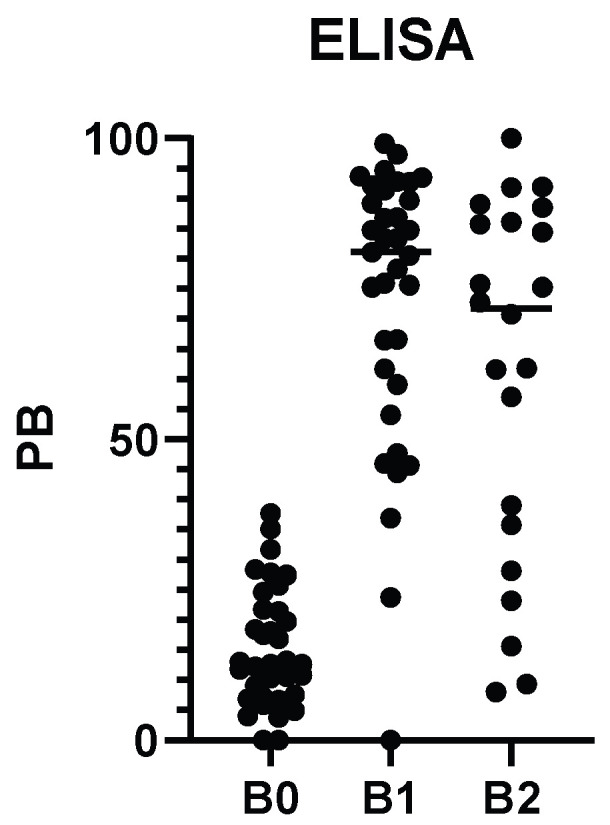
Box plot showing the levels of rVBA (ELISA) of the blood samples collected grouped per sampling point (values < 0% and >100% set at 0 and 100, respectively). Only dogs that tested seronegative for B0 are included (<40 PB); B0—day of vaccination, B1—30 dpv and B2—90 dpv.

A blood sample (B0) was collected immediately after bait consumption from 50 owned dogs that had been offered a bait. Eight dogs (16.0%) tested seropositive and were excluded from the follow-up study. Thirty days post-vaccination, 35 of the 42 dogs that had tested seronegative at baseline (B0) were relocated, and a second blood sample (B1) was collected. Of these dogs, 32 (91.4%, 95%CI: 79.3–97.6%) tested seropositive. A third blood sample (B2) was collected 90 dpv from 22 dogs that had originally tested seronegative at B0. Of these, 15 (68.2%, 95%CI: 48.5–84.0%) tested seropositive at 90 dpv. In total, 4 of these 22 dogs changed from seropositive to seronegative between the samples collected at 30 and 90 dpv. The seroconversion rate at 90 dpv (B2) was significantly lower than that observed at 30 dpv (B1) although the 95% confidence intervals overlapped; Chi-square = 5.05, df = 1, *p* = 0.025. Only 22 of the original 42 seronegative animals at baseline (B0) could be relocated 90 days later, indicating a high turnover within the dog population. Individual antibody levels (PB—percentage blocking) for the different blood samples that were originally seronegative dogs are presented in Figure 4.

## 4. Discussion

In this study, 98.4% of the dogs that consumed a bait were considered vaccinated. This proportion is extremely high compared with those reported in other studies using the same bait system. In these studies, the estimated vaccination rate among dogs that consumed an egg bait ranged from 65.7% to 95.2% [9,18,21]. However, in the bait acceptance studies conducted in Thailand, Indonesia and Morocco, the sachet contained blue-dyed water rather than a vaccine, and an animal was considered ‘vaccinated’ when the oral cavity, including the tongue, was visibly blue-stained. In the present study conducted in Cambodia, such an indicator for assessing vaccination success was not available. Instead, vaccination success was assessed indirectly on how the dog handled the bait and whether the sachet was perforated after being discarded. Therefore, the proportion of dogs that consumed the bait and were subsequently considered vaccinated may have been overestimated. Of all dogs approached and offered a bait, 66.4% were considered vaccinated. This value falls within the range reported in other studies using the same bait system, which varied from 51.5% (Morocco) to 75.4% (Bali) [12,18,19,20,21].

In contrast to these studies, only one bait type was used during the present study in Cambodia, the egg-flavored bait. Although this bait was relatively well accepted by dog populations in other countries in the region such as Bangladesh, Indonesia and Thailand, baits made from locally available materials (e.g., boiled cow or pork intestine segments) often achieved higher acceptance rates [10,12,18,27]. Similarly, in other studies, baits made from locally available materials frequently exhibited very high bait acceptance rates, including boiled intestine baits in the Philippines and the Navajo Nation, Köfte-bait (minced meat mixed with bread crumbs) in Türkiye, and chicken heads in Guatemala and Tunisia [19,28,29,30,31]. Numerous bait studies have been conducted in dogs and have demonstrated clear regional differences in bait preference, leading to the conclusion that there is no universally accepted bait for dogs [32]. Bait acceptance is influenced by many factors, including the familiarity of the local dog population with the bait material. Therefore, it has been suggested that the vaccine-filled sachets should preferably be incorporated into baits made from locally available material [32]. Unfortunately, this approach may result in highly variable efficacy among different vaccine bait types, as efficacy is determined not only by bait acceptance but also the immunogenic properties of the vaccine. To induce a protective immune response, the vaccine must be released into the oral cavity where it enters the host through the mucous membranes and subsequently undergoes limited replication, predominantly in the palatine tonsils [33]. Thus, effective bait uptake by dogs is also influenced by the shape, size and texture of both the bait and sachet. For example, the Köfte bait used in Türkiye was extremely well accepted by the local dogs; however, the vaccine-containing sachet was often swallowed without being perforated. Consequently, the vaccine was not released in the oral cavity, resulting in vaccination failure [31]. It has been shown that the difference between the acceptance rate of the egg-flavored bait and the subsequent release rate of the sachet contents into the oral cavity is relatively small compared to, for example, boiled intestine baits [10,18,20,21]. Therefore, an industrially manufactured, standardized bait system including both the bait matrix and sachet, with proven efficacy in dogs, such as the egg-flavored vaccine bait is likely to provide more reliable results.

The significant effect of the factor ‘date’ on the proportion of dogs showing interest in and subsequently consuming the baits offered in this study is difficult to explain. In other studies, an effect of increasing experience has occasionally been observed. During successive days, field teams may become more experienced and therefore more effective at approaching dogs and offering baits [20]. In the present study, however, such a ‘learning’ effect was not observed. The daily fluctuations observed may instead have been caused by factors such as prevailing weather conditions. Another interesting observation was that dogs showed less interest in the baits during the early morning. It is often assumed that dogs show little interest in food during the hottest periods of the day and that, therefore, baits should be offered during the cooler periods, preferably early morning. However, most owned dogs in this study were fed early in the morning, which could explain the reduced interest in the baits. Another possible explanation for the relatively poor uptake during the early morning may be the low temperature of the baits. Baits were stored frozen, thawed overnight in a refrigerator, and subsequently transferred to cool boxes in the early morning. The relatively low bait temperatures during this period of the day may negatively affect the smell and taste of the baits, possibly resulting in reduced bait uptake (Vos, personal observation). When a dog was offered a bait in the presence of other dogs, the consumption rate was higher than when the dog was alone. This phenomenon has also been observed in other studies; when dogs are together and one starts eating, the other dogs are more likely to consume the bait offered. The observation that smaller dogs accepted and consumed baits more frequently may also be influenced by age. Young animals, most often small-sized, tend to accept baits more readily than adult dogs.

Eight dogs tested seropositive although their owners claimed that the animals had not been vaccinated against rabies. False-positive results are very uncommon with this assay: In experimental studies involving truly naïve dogs, only 1 of 110 animals tested seropositive [11,34]. Hence, there is good reason to believe that the findings obtained from pre-treatment sera in this study conducted in Cambodia represent true positive results. Two explanations may be proposed: (1) dogs reported as unvaccinated had in fact been vaccinated previously, or (2) naturally acquired immunity has developed following prior exposure to RABV without progression to clinical disease. The latter explanation is not yet widely accepted; however, it cannot be ruled out, as rabies-specific antibodies have been detected in healthy, unvaccinated individuals from a variety of domestic dog populations and wildlife reservoir hosts in rabies-endemic areas, with a wide range of seroprevalence values [35,36,37,38,39].

The obtained seroconversion rate (91.4%) 30 dpv was slightly higher than those observed in similar field studies using the same vaccine bait and assay, which ranged from 76.2% to 90.9% [17,20,22]. The significant decline to 68.2% seroconversion at 90 dpv was unexpected, as both experimental and field studies have shown that SPBN GASGAS induces long-lasting immunity, with no significant decrease in seroconversion rates for up to 3 years post-vaccination [20,21,34,40]. The relatively small sample size at 90 dpv, resulting from an exceptionally high loss to follow-up, may have contributed to this apparent effect, particularly since the 95% confidence intervals of the seroconversion rates at 30 and 90 dpv still overlapped.

Several limitations were identified. First, the impact of data exclusion should be considered. The reason why so many contradictory data entries were recorded remains unknown. Although the vaccination teams were familiar with electronic data collection, they lacked experience with bait acceptance studies and may therefore have misinterpreted certain questions. In future studies, more intensive training could help prevent such errors. In addition, daily review of the collected data would facilitate the timely implementation of corrective measures and reduce the number of erroneous entries. Furthermore, the unexpectedly high loss at follow-up during the immunogenicity study reduced the sample size at 90 dpv to below the minimum required sample size. Finally, as previously mentioned, the vaccination rate estimated during the bait acceptance study may have been overestimated.

## 5. Conclusions

Recently, the beneficial role of ORV of dogs has been emphasized by the global rabies community [32,41,42] and the present study further demonstrated the practicability of this approach in Cambodia, with its large population of owned yet free-roaming dogs. The bait acceptance and vaccine-induced immunogenicity observed following bait consumption in Cambodia were comparable to those observed from other countries. However, ORV is only part of the solution. The elimination of dog-mediated rabies in Cambodia will require coordinated and effective management strategies involving not only the veterinary sector but also the public health sector, local stakeholders and dog owners.

## Figures and Tables

**Table 1:** Summary of selected bait and sachet parameters in relation to the estimated vaccination rate. The ‘unknown’ data entries were not used for the calculations of percentages and thus not listed here.

	Amount of Bait Consumed		‘Vaccinated’ ^1^
	N	%	(%)
100%	321	69.8	98.8
>50%	126	27.4	97.6
<50%	13	2.8	91.7
	**Duration of Bait Consumption (s)**		**‘Vaccinated’**
	N	%	(%)
>60	29	6.3	100
31–60	197	42.8	99.5
10–30	123	26.7	95.9
<10	111	24.1	98.2
	**Sachet**		**‘Vaccinated’**
	N	%	(%)
discarded	446	970	97.8
swallowed	14	3.0	42.9
	**Sachet Perforation ^2^**		**‘Vaccinated’**
	N	%	(%)
Yes	442	98.4	99.8
No	7	1.6	0

1—‘vaccinated’—release of vaccine in oral cavity as determined by direct observation. 2—for 11 sachets, it was not known if they were perforated or not.

**Table 2:** Summary of the measured responses (bait interest, bait consumption, vaccination success) for the different settings of the listed factors. Data recorded as ‘unknown’ are not included in this analysis; furthermore, for the calculations for the response consumed and vaccinated, only the number of dogs shown interest and number of dogs that consumed the baits are included, respectively.

Factor	Settings	Response 1 Interested n/N (%)	Response 2 Consumed n/N (%)	Response 3 ‘Vaccinated’ ^(5)^ n/N (%)
date		Chi^2^ = 59.67, df = 6, *p* < 0.0001	Chi^2^ = 46.52, df = 6, *p* < 0.0001	Chi^2^ test not valid
	12 February 2024	63/72 (87.5%)	53/63 (84.1%)	49/52 (94.2%)
	13 February 2024	90/125 (72.0%)	84/90 (95.5%)	84/84 (100%)
	14 February 2024	40/75 (53.3%)	40/40 (100%)	40/40 (100%)
	15 February 2024	91/140 (65.0%)	78/90 (86.7%)	78/78 (100%)
	16 February 2024	55/76 (72.4%)	41/55 (74.6%)	38/41 (92.7%)
	21 February 2024	39/47 (83.0%)	29/39 (74.4%)	27/29 (93.1%)
	25 February 2024	137/148 (92.6%)	135/137 (98.5%)	135/135 (100%)
time		Chi^2^ = 19.52, df = 7, *p* = 0.0067	Chi^2^ test not valid	Chi^2^ test not valid
	08:00–08:59	14/24 (58.3%)	10/14 (71.4%)	7/8 (87.5%)
	09:00–09:59	79/110 (71.8%)	63/78 (80.8%)	61/63 (96.8%)
	10:00–10:59	99/131 (75.6%)	94/99 (95.0%)	92/94 (97.9%)
	11:00–11:59 ^(2)^	81/99 (81.8%)	76/81 (93.8%)	74/75 (98.7%)
	14:00–14:59 ^(3)^	96/117 (82.1%)	92/86 (95.8%)	92/92 (100%)
	15:00–15:59	86/101 (85.2%)	77/85 (90.6%)	76/77 (98.7%)
	16:00–16:59	53/58 (91.4%)	41/52 (78.8%)	40/41 (97.6%)
	17:00–17:59 ^(4)^	7/9 (77.8%)	7/7 (100%)	7/7 (100%)
social		Chi^2^ = 7.305, df = 1, *p* = 0.0069	Chi^2^ = 31.17, df = 1, *p* < 0.0001	Chi^2^ = 1.460, df = 1, *p* = 0.2269
	alone	178/217 (82.0%)	140/176 (79.6%)	136/140 (97.1%)
	multiple	337/465 (72.5%)	320/336 (95.2%)	315/319 (98.8%)
size ^(1)^		Chi^2^ = 17.23, df = 1, *p* < 0.0001	Chi^2^ = 1.606, df = 1, *p* = 0.2051	Chi^2^ = 2.209, df = 1, *p* = 0.1372
	large	6/7 (85.7%)	5/6 (83.33%)	5/5 (100%)
	medium	400/555 (72.1%)	355/399 (89.0)	346/354 (97.74%)
	small	104/115 (90.4%)	96/103 (93.2%)	96/96 (100%)
sex		Chi^2^ = 0.1190, df = 1, *p* = 0.7301	Chi^2^ = 3.970, df = 1, *p* = 0.0463	Chi^2^ = 2.114, df = 1, *p* = 0.1459
	male	329/439 (74.9%)	289/329 (87.8%)	281/288 (97.6%)
	female	185/243 (76.1%)	170/182 (93.4%)	169/170 (99.4%)

(1) For the statistical analysis, the group ‘large’ has not been included due to the small sample size. (2) Two data entries with times 12:01 and 12:03 were included in this subgroup. (3) Four data entries with times 13:51 (3x) and 13:52 were included in this subgroup. (4) Two date entries with times 18:00 and 18:01 were included in this subgroup. (5) ‘vaccinated’—release of vaccine in oral cavity as determined by direct observation.

## Data Availability

The original data can be provided upon reasonable request and should be directed to the corresponding author.
